# Individual differences in face perception and person recognition

**DOI:** 10.1186/s41235-018-0109-4

**Published:** 2018-06-27

**Authors:** Vicki Bruce, Markus Bindemann, Karen Lander

**Affiliations:** 1 Newcastle University, Newcastle, UK; 2 University of Kent, Canterbury, Kent, England; 3 University of Manchester, Oxford Rd, Manchester, England

Cognitive Research: Principles and Implications has now released the first batch of articles on this special topic. In addition to this editorial, we (Lander, Bruce & Bindemann, 2018) have published here a narrative review of the topic. In our review we note that, with the exception of work on impairments in face recognition (prosopagnosia), research has only recently begun to investigate why there are such wide variations in individual abilities to perceive and recognise faces. These investigations have raised as many questions as answers about the reasons why some people are so much better than others at recognising faces. Our review also highlighted two specific areas of application - the recruitment and use of “super-recogniser” (SRs) in forensic operations, and the scrutiny of passport or other identity photographs used to gain access to restricted areas. These are areas that a number of the papers published here address.

McCaffery, Robertson, Young & Burton (2018) measure performance on a test of face comparison, the Glasgow Face Matching Test (hereafter GFMT), a test of face memory, the Cambridge Face Memory Test (hereafter CFMT) and a test of recognising familiar faces “Before They Were Famous” (BTWF). They investigate correlation between performance on the different tests and correlation between the tests and self-assessment of face-recognition ability (in a first study) and other perceptual matching and recognition tasks (in a second study). In general, there was correlation between the face tasks, consistent with the idea that there is a general face-perception factor, which appears to account for about 25% of performance variance (cf. Verhallen et al., 2017). Task-specific influences were also found - e.g., people’s self-ratings of face-recognition ability correlated only with BTWF and non-face tasks that required matching correlated only with GFMT.

Thus, McCaffery et al. reinforce evidence that some people are better than others at a range of face perception and recognition tasks and that such facility cannot be attributed entirely to more general perceptual or memory abilities. While such differences appear to support the identification and recruitment of SRs, the paper by Sarah Bate and colleagues (2018) suggests that more complex, task-specific screening tools may be needed. They recruited 200 people who thought they were potential SRs and tested them on the long form of the CFMT and three new and demanding tests of face matching, face memory and searching crowds for faces resembling a composite image. While a (bare) majority of the 200 showed some degree of consistently good performance across two or more tests, fewer than 50% of them (89 in total) performed well enough on the CFMT alone to support their self-assessment as SRs. And of these, just 37 were also superior at both the other tests of face memory and matching. Performance on the new test of matching to crowds was not predicted by any of the other tests.

Megan Papesh (2018) adds to previous research (e.g., White, Kemp, Jenkins, Matheson, & Burton, 2014) by showing that professionals, whose jobs require frequent image-matching, are no better than inexperienced student control participants at matching identities between face images. She recruited over 800 professional notaries and 70 bank tellers and found that they were no better than undergraduate controls at a face-matching task. Moreover, individual differences in the frequency of face matching in these occupational settings, and years of work experience, did not impact on the professionals’ performance. However, performance was negatively correlated with age, with older participants performing more poorly.

Where scrutinising facial identities is an important component of a job, there may also be scope to recruit people likely to perform more accurately. Balsdon, Summersby, Kemp & White (2018) evaluated the efficacy of using screening tests to select for the job of scrutinising submitted passport photographs for validity. There was correlation between performance on the three screening tests used (CFMT, GFMT and a self-report questionnaire), but selecting people who scored at the top end of such tests as potential passport image-checkers yielded only modest gains in the authors’ real-world fraud detection test. In contrast, however, pooling decisions from two or more image-checkers led to much more substantial gains, showing that in difficult image-matching tests, using the “wisdom of crowds” approach may be a fruitful way to circumvent problems of human (and machine) error.

The job of checking passport images may become still more challenging as newer methods of fraud become deployed. For example, Robertson et al. (2018) describe how a criminal could morph their own image with that of a genuine passport-holder (whose document may have been stolen or may belong to a confederate) and use the morphed image in an application for a passport renewal. The morphed image could match that previous one held by the government well enough to generate a genuine but fraudulently obtained passport. This in turn would sufficiently resemble the criminal to pass detection at a border. Robertson et al. show that there are individual differences in people’s abilities to detect “morphed” faces, and that people can benefit from training on this task. After training, they identified significant correlation between detecting morphed images and detecting mismatches in a difficult (non-morphed) face-matching task. Like other papers on this special topic, and previous research too (e.g., Kokje, Bindemann, & Megreya, 2018; Megreya & Burton, 2007) this illustrates how verifying matches and detecting mismatches may involve different skills.

A different kind of fraud can arise from the use of hyper-realistic face masks, as described in the paper by Sanders & Jenkins (2018). Here again, the authors show that there is wide individual variation in ability to spot such fake faces, and here there is no correlation with other face-matching abilities. Examination of what makes some people better than others at this suggests that reliance on local information around the eyes is key to this task, demonstrating that some very specific sub-skills may underlie certain real-world applications.

While faces may be the most important key to identity, in many everyday situations there may be information available from bodies as well. An eye witness to a crime remembers more than just the face of a criminal - they will describe their height, build and perhaps gait too. Noyes, Hill & O’Toole (2018) investigate whether screening with the GFMT predicts performance on matching faces (in a different task), matching bodies and matching bodily motions from point-light displays. Although groups identified as “good” or “bad” face matchers do also differ on performance in matching bodies, examination of individual differences showed that the GFMT correlates only with the other face-matching test, and not with the two body-matching tests. This underlines that for practical screening and/or theoretical interpretation the analysis of individual differences is essential. Noyes et al. argue that this “points to the use of individual differences to inform how or, indeed, whether to apply group analyses” rather than to individual differences being mentioned only as an afterthought.

There is thus considerable evidence that there is correlation between different tasks of face matching and memory. Some evidence for this correlation was also observed by Matthew Fysh in a further paper on this issue (2018). However, performance on tasks that tap aspects of face identification was not correlated with performance on a task of detecting faces, demonstrating further differentiation of face-related abilities.

We anticipate further papers will join this special topic as revisions of more articles are accepted over the next few weeks. We expect papers on the topic of individual differences and/or selection of eyewitnesses in line-ups will join the collection surveyed here.
